# Network Analysis of Food Intake Patterns and Frailty Dimensions in Chinese Older Adults: A National Cross-Sectional Study

**DOI:** 10.3390/nu18142310

**Published:** 2026-07-14

**Authors:** Jinlu Bian, Weitong Li, Hui Shi, Luoru Ding, Run Ye, Guihua Xu

**Affiliations:** 1 School of Nursing, Nanjing University of Chinese Medicine, Nanjing 210023, China; 202511182@njucm.edu.cn (J.B.); nzylwt@njucm.edu.cn (W.L.); 19551299881@163.com (H.S.); 15312712226@163.com (L.D.); 2 School of Physics and Electronic Engineering, Yancheng Teachers University, Yancheng 224007, China

**Keywords:** frailty, food intake pattern, older adults, network analysis

## Abstract

**Background**: Frailty is a public health concern in older adults. However, associations between food intake patterns and frailty dimensions remain insufficiently understood. This study aimed to construct a network linking food intake patterns with frailty dimensions, identify nodes with high centrality and bridge nodes, and provide evidence for frailty identification and factors that may be associated with frailty in older adults. **Methods**: We included 14,140 older adults from the 2017–2018 Chinese Longitudinal Healthy Longevity Survey (CLHLS). Food intake was assessed using a validated food frequency questionnaire, and patterns were extracted by exploratory factor analysis. Frailty was assessed using a frailty index based on 35 health-related deficits and classified into three dimensions: basic status (BS), basic ability (BA), and medical history (MH). Network analysis with a Gaussian graphical model was used to construct a partial correlation network and identify central and bridge nodes. **Results**: Four food intake patterns were identified: FD-1 (legumes–eggs–milk pattern), FD-2 (fruits–vegetables pattern), FD-3 (garlic–nuts–salt-preserved vegetables pattern), and FD-4 (meat–fish pattern). FD-1 and FD-2 showed high strength centrality, indicating strong connections within the network. The strongest cross-network association was negative between FD-2 and BS. FD-3 was negatively associated with BA, whereas FD-4 was weakly positively associated with MH. BA had the highest bridge strength, while FD-1 showed high bridge closeness and betweenness. **Conclusions**: Food intake patterns showed complex associations with frailty dimensions. FD-1, FD-2, and BA emerged as potential nodes of interest. Future studies are warranted to investigate whether dietary strategies centered on FD-1 and FD-2 are associated with preserved function and reduced frailty risk in older adults.

## 1. Introduction

Across the globe, the proportion of older adults is increasing. By the end of 2025, the number of people aged 60 and above in China reached 323.38 million, accounting for 23.0% of the total population; the number of people aged 65 and above in China reached 223.65 million, accounting for 15.9%, and the problem of population aging has further intensified [1]. Frailty is an extreme consequence that occurs during the normal aging process of the human body, and it is also the most severe form of population aging [2]. Frailty is a vulnerable state characterized by the continuous decline of the physiological system and a decreased ability to resist stressors [3]. A systematic review and meta-analysis revealed that the overall prevalence of frailty and frailty-related conditions among community-dwelling older adults in China was 10.1% and 43.9%, respectively [4]. Frailty can increase the risks of falls, fractures, hospitalizations, and deaths [5,6]. It not only imposes a burden on older adults themselves, their families, and caregivers, but also puts pressure on the medical and social care systems [7]. Therefore, early identification of frailty in older adults can help identify the risk factors and formulate precise measures potentially associated with frailty, thereby reducing the burden on the healthcare system and promoting successful aging.

Frailty is closely related to unhealthy lifestyles and food intake patterns [8,9]. A study conducted by Qi et al. revealed that appropriate dietary behaviors play a crucial role in influencing the risk of frailty among older adults [10]. The research conducted by Mendonça et al. indicated that higher protein intake moderately reduced the risk of frailty and may, in a dose-dependent manner, increase the likelihood of middle-aged and older adults recovering from the pre-frailty stage [11]. Adhering to the Mediterranean diet was associated with a reduction in frailty among older adults [12,13]. A prospective study conducted by Huang et al. found that compared to an omnivorous diet, a vegetarian diet might be associated with a higher risk of frailty [14]. A recent study has shown that increasing the intake of fruits and vegetables was associated with a lower rate of frailty [15]. The research conducted by Zhang et al. indicated that starting a diverse diet as early as possible and maintaining it over a long period could reduce the risk of frailty [16]. The research conducted by Tanaka et al. indicated that a diet rich in plant proteins was associated with a reduction in the degree of frailty [17]. However, previous studies on frailty merely focused on and measured the overall dietary score, without fully considering the various dimensions of frailty and their relationship with different food intakes. Furthermore, exploratory factor analysis (EFA) can identify specific food intake patterns. Previous studies have used this method to identify different food intake patterns and explore their relationships with various health outcomes, such as depression in older adults [18], the risk of hyperuricemia [19], and anemia [20]. In contrast, the food intake pattern is more in line with real dietary behaviors. People usually consume a combination of various foods rather than a single nutrient or food. Due to the potential interactions and synergistic effects among the components of the food intake pattern, such patterns may be more effective in controlling the frailty of older adults [16,21,22,23].

In recent years, network analysis has been widely applied in the field of geriatric health. The core components of network analysis include nodes and edges. The central node usually refers to the node with stronger connections and greater influence in the network, while the bridge node reflects the connection role between different variable modules [24]. In a study on the relationship between food intake and cognitive function in older adults with multiple diseases, network analysis identified the core nodes (mushrooms or algae, dairy products, nut products) and bridge nodes (language ability, orientation ability, nut products) in the food intake and cognitive function network, clarifying the correlation characteristics between them. This provided a scientific basis for targeted dietary interventions to improve cognitive function in older adults and individuals with multimorbidity [25]. Another study demonstrated that through the construction of a network model for depression and anxiety symptoms among older adults living alone in China, it identified core symptoms such as “uncontrollable worry” and “difficulty in relaxation”, as well as bridging symptoms like “tension or anxiety” and “feeling depressed”. This revealed complex interconnections among symptoms and sex differences, providing support for nodes of interest to guide interventions [26]. However, at present, there is a lack of network analysis studies on the food intake patterns and frailty in older adults.

Therefore, this study employed network analysis to construct a network model between food intake patterns and frailty dimensions among Chinese older adults. Nodes with high centrality and bridge nodes were identified to clarify the complex relationship between the food intake patterns of older adults and frailty, and to identify potential dietary correlates that may inform future hypothesis-driven research.

## 2. Materials and Methods

### 2.1. Study Population

The Chinese Longitudinal Healthy Longevity Survey (CLHLS) is a nationwide longitudinal study of older adults, conducted by the Center for Healthy Aging and Development Studies, National School of Development of Peking University. The survey covers 23 provinces, municipalities, and autonomous regions across China and employs a multi-stage, stratified, cluster sampling method, targeting individuals aged 65 and above. Trained investigators conducted face-to-face household interviews using standardized questionnaires, collecting data on basic demographics, socioeconomic status, health and quality of life, cognitive function, and activities of daily living. The baseline survey was launched in 1998, and eight waves of data collection had been completed as of 2017–2018. Detailed descriptions of the CLHLS research design, sampling methods, and data quality are available in references [27,28,29,30,31]. CLHLS received ethical approval from the Peking University Biomedical Ethics Committee (IRB00001052-13074), and all participants or their proxies signed informed consent forms.

**Figure 1:**
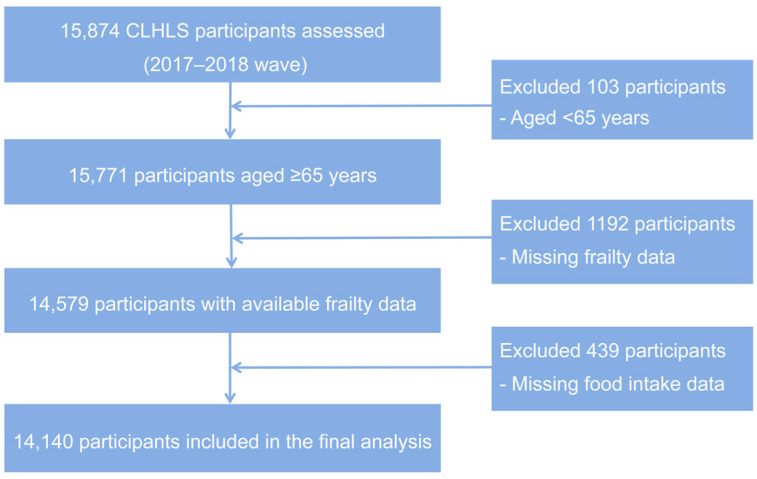
Flowchart of study participant selection.

This study used data from the 8th wave of the CLHLS conducted in 2017–2018. This round represented one of the most recent nationwide survey cycles, having interviewed 15,874 individuals aged 65 and older, providing a large sample size capable of comprehensively reflecting the health status of China’s older adult population. We excluded participants who were under 65 years old at the time of the survey (103 individuals), lacked frailty information (1192 individuals), or had missing food intake data (439 individuals), resulting in a final analytical sample of 14,140 older adults. The participant selection process is shown in Figure 1.

### 2.2. Assessment of Food Intake

This study used a simplified food frequency questionnaire (FFQ) to assess food intake [25,32], including the types and frequencies of foods consumed. This study included 14 of the most common food groups in the Chinese diet: whole grains, vegetable oil, fresh fruits, fresh vegetables, legumes, garlic, nuts, tea, salt-preserved vegetables, sugar, meat, fish and other aquatic products, eggs, and milk products. The scoring rules for the FFQ were as follows: whole grains and plant oils (no = 0, yes = 1); fresh fruits and vegetables (daily/almost daily = 4, frequently = 3, sometimes = 2, rarely or never = 1); the remaining 10 food groups (almost daily = 5, not daily but at least once per week = 4, not weekly but at least once per month = 3, not monthly but occasionally = 2, rarely or never = 1) (Scoring details are provided in Supplementary Table S1). It should be noted that the FFQ contains both binary and ordinal scoring scales across food groups, which may introduce heterogeneity in measurement scale across dietary variables.

### 2.3. Frailty Assessment

In 2001, the concept of the frailty index (FI), based on the cumulative deficit theory, was introduced [33]. This study constructed the FI in accordance with established standards [34]. Based on previous studies [10,35], this study constructed the FI using 35 health-related indicators and divided it into three dimensions: BS (basic status), BA (basic abilities), and MH (medical history). (The indicators are listed in Supplementary Table S2).

Each health deficit indicator was assigned a score based on the severity of the deficit. When a health indicator had two response options, scores were assigned as 0 and 1; for three options, scores were 0, 0.5, and 1; for four options, scores were 0, 0.33, 0.67, and 1; and for five options, scores were 0, 0.25, 0.5, 0.75, and 1. Here, 0 indicated normal status, and 1 indicated the presence of a deficit. Individuals with fewer than 25 available health indicators were excluded from analysis. The FI score was calculated by dividing the number of health deficit indicators present in an individual by the total number of health indicators actually measured. The score ranged from 0 to 1, with higher scores indicating greater frailty. In this study, FI was categorized into three groups: FI ≤ 0.10 indicated no frailty, 0.10 < FI ≤ 0.21 indicated pre-frailty, and FI > 0.21 indicated frailty [10,36,37].

### 2.4. Covariates

Based on previous studies [4,38,39,40,41], several common factors influencing frailty were selected as covariates. The covariates included age (in years), sex (male, female), place of residence (urban, rural), economic status (affluent, moderate, or deprived), living arrangement (not living alone, living alone), marital status (married or living with partner or separated, divorced or widowed or never married), smoking status (smoker, non-smoker), drinking status (drinker, non-drinker), physical exercise (exerciser, non-exerciser), and BMI (body mass index). BMI was calculated as weight (kg) divided by height (m)^2^ and further categorized into underweight, normal weight, overweight, and obese.

### 2.5. Statistical Analysis

Statistical analysis was performed using R software (version 4.5.1). First, data were preprocessed, including handling missing values and standardizing variables for network analysis. Descriptive statistics were used to summarize the basic characteristics of the study participants: categorical variables were presented as frequencies and percentages, while continuous variables were expressed as means and standard deviations. Participants were classified into three groups based on frailty status—non-frailty, pre-frailty, and frailty. Categorical variables were compared across groups using chi-square tests, and continuous variables were analyzed using analysis of variance (ANOVA). All statistical tests were two-tailed, with a significance level set at *p* < 0.05.

Exploratory factor analysis was used to extract food intake patterns, and the composition of each pattern was determined based on factor loadings. The number of factors to retain was determined based on multiple criteria: Kaiser’s criterion (eigenvalues > 1), parallel analysis, visual inspection of the scree plot, and interpretability of the factor structure. The Kaiser–Meyer–Olkin (KMO) measure and Bartlett’s test of sphericity were used to confirm the suitability of the data for factor analysis. Factors with loadings ≥ 0.30 were considered meaningful for pattern interpretation. Following previous studies [10,35] and the content of the frailty index indicators, the 35 health-related deficit indicators were categorized into three dimensions: BS (basic status), BA (basic abilities), and MH (medical history). Cumulative scores for each dimension were calculated and used as frailty dimension nodes in network analysis; the FI score was used to group participants according to their frailty status.

A Gaussian graphical model (GGM) was used to construct a network linking food intake patterns and frailty dimensions [42], visualized using the Fruchterman–Reingold algorithm [43,44]. Nodes represented food intake patterns or frailty dimensions, while edges indicated partial correlations between two nodes after controlling for all other nodes in the network. Edge color reflected the direction of correlation, and edge thickness represented the strength of association. To reduce spurious correlations, graphical LASSO (glasso) regularization was applied [45], and the extended Bayesian information criterion (EBIC) was used to select the optimal regularized network [42].

Node importance was assessed using centrality measures, including strength, closeness, and betweenness. Bridge centrality analysis was conducted to evaluate cross-module connections between food intake pattern modules and frailty dimension modules, with indicators including bridge strength, bridge closeness, and bridge betweenness. The bootnet package was employed to assess the stability and accuracy of the network models: the case-dropping bootstrap was used to compute the correlation stability coefficient (CS), and the non-parametric bootstrap was used to evaluate the accuracy of edge-weight estimation. Generally, CS > 0.25 indicates that the minimum stability requirement is met, CS > 0.50 indicates moderate stability, and CS > 0.70 indicates high stability [25,46]. Finally, separate network models were constructed for male and female older adults, and the above procedures—including network estimation, centrality analysis, bridge centrality analysis, and stability assessment—were repeated independently for each group.

To evaluate the robustness of our network findings to the number of factors retained in the exploratory factor analysis, we conducted a sensitivity analysis by re-estimating the Gaussian graphical model using 3-factor and 5-factor solutions in addition to the 4-factor solution. The centrality rankings of the dietary pattern nodes (specifically FD-1 and FD-2) were compared across the three solutions to assess whether the main conclusions were stable under alternative factor retention assumptions.

## 3. Results

### 3.1. Description of Characteristics

This study included a total of 14,140 participants with a mean age of 85.33 ± 11.57 years, including 3037 (21.5%) aged < 75 years and 11,103 (78.5%) aged 75 or older. There were 6214 males (43.9%) and 7926 females (56.1%). Most participants resided in rural areas (*n* = 10,062, 83.2%), while 2033 (16.8%) lived in urban areas, with 2045 participants having missing data on residence status. Based on frailty status, the distribution was as follows: non-frailty (*n* = 3072, 21.7%), pre-frailty (*n* = 6552, 46.3%), and frailty (*n* = 4516, 31.9%). Inter-group comparisons revealed significant differences among the different frailty statuses in terms of age group, sex, economic level, cohabitation status, marital status, smoking, drinking, physical exercise, and BMI category (all *p* < 0.05), as shown in Table 1.

### 3.2. Network Structure Analysis

The KMO measure is 0.76, indicating acceptable sampling adequacy for factor analysis (values > 0.70 are considered acceptable, >0.80 good), and the item-level Measures of Sampling Adequacy (MSA) are presented in Supplementary Table S3. In addition, Bartlett’s test of sphericity was significant (χ^2^ = 1335.075, *df* = 91, *p* < 0.001), confirming the suitability of the data for factor analysis. Parallel analysis and scree plot inspection both supported the retention of four factors (Supplementary Figure S1). The four-factor solution explained 58.0% of the total variance. Factor loadings for each food item are presented in Supplementary Table S4.

Exploratory factor analysis identified four distinct food intake patterns: food intake pattern 1 (FD-1), including legumes, eggs, and milk; food intake pattern 2 (FD-2), including fruits and vegetables; food intake pattern 3 (FD-3), including garlic, nuts, and salt-preserved vegetables; and food intake pattern 4 (FD-4), including meat and fish.

**Figure 2:**
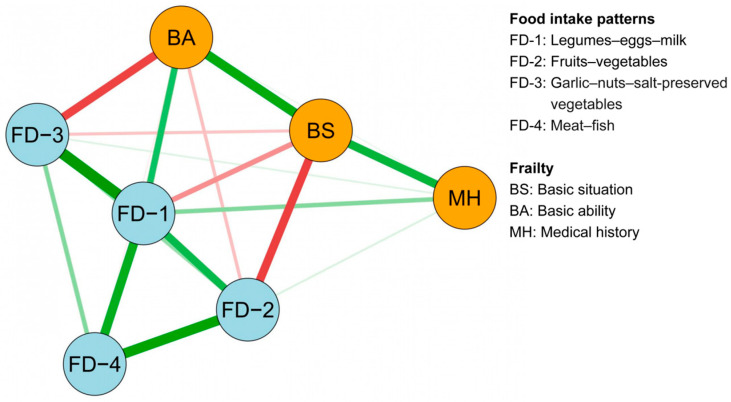
Network structure of food intake patterns and frailty among overall older adults. Nodes represent food intake patterns or frailty dimensions; edges indicate partial associations after controlling for other nodes. Green edges represent positive correlations, red edges represent negative correlations; edge thickness indicates the strength of association. The network layout was generated using the Fruchterman–Reingold force-directed algorithm.

Figure 2 illustrates the network relationships between food intake patterns and frailty dimensions among the overall older adult population. Among the connections between food intake patterns and frailty dimensions, FD-2 (fruits–vegetables pattern) showed the strongest association with the frailty node BS (basic status), exhibiting a negative correlation, followed by FD-3 (garlic–nuts–salt-preserved vegetables pattern) and the frailty node BA (basic ability), also showing a negative correlation. The association between FD-4 (meat–fish pattern) and MH (medical history) was weaker and positively correlated. In the food intake pattern subnetwork, FD-1 (legumes–eggs–milk pattern) had the strongest positive correlation with FD-3 (garlic–nuts–salt-preserved vegetables pattern), followed by the positive correlation between FD-2 (fruits–vegetables pattern) and FD-4 (meat–fish pattern). In the frailty dimension subnetwork, BA (basic ability) showed the strongest positive correlation with BS (basic status), followed by the positive correlation between MH (medical history) and BS (basic status) (correlation matrix in Supplementary Table S5; network diagram for the overall older adult population shown in Figure 2).

The figure illustrates the food intake patterns stratified by sex among older adults and their associations with frailty dimensions. In the male network (correlation matrix shown in Supplementary Table S6), FD-2 (fruits–vegetables pattern) showed the strongest negative association with the frailty node BS (basic status). FD-1 (legumes–eggs–milk pattern) was most strongly positively correlated with FD-3 (garlic–nuts–salt-preserved vegetables pattern), while the frailty node BA (basic ability) exhibited the strongest positive correlation with BS (basic status), consistent with findings in the overall older adult population (male older adults network shown in Supplementary Figure S2A). In the female network (correlation matrix shown in Supplementary Table S7), FD-2 (fruits–vegetables pattern) also showed the strongest negative association with the frailty node BS (basic status). FD-2 (fruits–vegetables pattern) had the strongest positive correlation with FD-4 (meat–fish pattern), and the frailty node BA (basic ability) was most strongly positively associated with BS (basic status), showing slight differences compared with the overall older adult group (female older adults network shown in Supplementary Figure S2B).

### 3.3. Centrality Analysis

**Figure 3:**
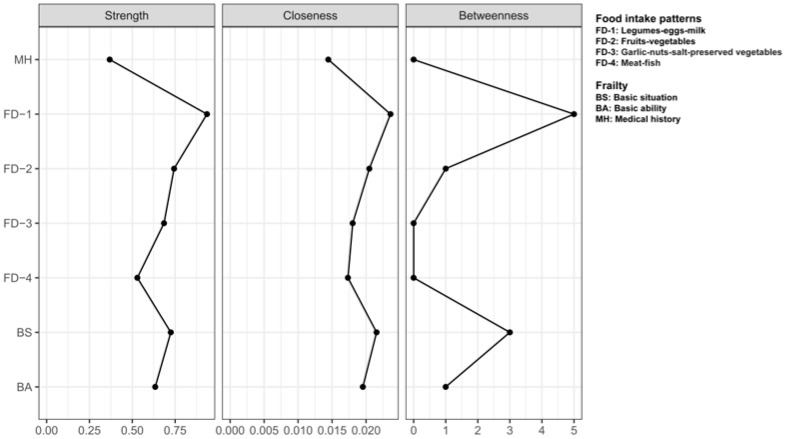
Centrality indicators for the food intake patterns and frailty network among overall older adults.

Among network centrality measures, strength centrality quantifies the total direct connections of a node to other nodes and reflects its overall importance within the network. Closeness centrality indicates how close a node is to all other nodes in the network, representing its efficiency in disseminating information across the entire network. Betweenness centrality measures the extent to which a node acts as a bridge along the shortest paths between other nodes, reflecting its capacity to regulate information flow. In the overall older adult population, FD-1 (legumes–eggs–milk pattern, strength = 0.936) and FD-2 (fruits–vegetables pattern, strength = 0.744) exhibited relatively high strength values among all nodes in the full network model. Regarding closeness centrality, FD-1 (legumes–eggs–milk pattern, closeness = 0.024) and BS (basic status, closeness = 0.022), a frailty-related node, showed higher values. For betweenness centrality, FD-1 (legumes–eggs–milk pattern, betweenness = 10) and BS (basic status, betweenness = 6) also ranked higher. Among the nodes in the food intake pattern model, FD-1 (legumes–eggs–milk pattern, strength = 0.936, closeness = 0.024, betweenness = 10) and FD-2 (fruits–vegetables pattern, strength = 0.744, closeness = 0.020, betweenness = 2) demonstrated relatively high scores across strength, closeness, and betweenness centralities. Within the frailty dimension, the frailty-related nodes BS (basic status, strength = 0.725, closeness = 0.022, betweenness = 6) and BA (basic abilities, strength = 0.634, closeness = 0.020, betweenness = 2) also showed relatively high centrality indicators (Figure 3 shows the centrality indicators for the overall older adult population; specific numerical values are provided in Supplementary Table S8). In sex-stratified analyses, among male older adults, FD-1 (legumes–eggs–milk pattern, closeness = 0.023) and FD-2 (fruits–vegetables pattern, closeness = 0.022) had higher closeness centrality values in the full network model, while nodes with high strength and betweenness centrality were consistent with those observed in the overall older adult population (centrality indicators for male older adults shown in Supplementary Figure S3A; specific values in Supplementary Table S9). Similarly, among female older adults, the nodes with high strength, closeness, and betweenness centralities were consistent with those in the overall older adult population (centrality indicators for female older adults shown in Supplementary Figure S3B; specific values in Supplementary Table S10).

### 3.4. Bridge Centrality Analysis

Among the overall older adult population, in the full network model, BA (basic ability) exhibited the highest bridge strength (bridge strength = 0.410). FD-1 (legumes–eggs–milk pattern, bridge closeness centrality = 0.101, bridge betweenness centrality = 9) ranked highest in both bridge closeness centrality and bridge betweenness centrality. Among nodes in the food intake pattern, FD-1 (legumes–eggs–milk pattern, bridge strength = 0.347, bridge closeness centrality = 0.101, bridge betweenness centrality = 9) showed the highest values for bridge strength, bridge closeness, and bridge betweenness centrality. In the frailty dimension model, the node BA (basic abilities, bridge strength = 0.410, bridge closeness = 0.092, bridge betweenness = 4) had the highest scores across all three bridge centrality measures (specific values of bridge centrality indicators for the overall older adult population are shown in Supplementary Table S11). The nodes with the highest values for the three bridge centrality measures in the sex-stratified subgroups were consistent with those observed in the overall older adult population across the full network model, food intake pattern model, and frailty model. (Specific values of bridge centrality indicators for male and female older adults are presented in Supplementary Tables S12 and S13, respectively).

### 3.5. Network Stability

**Figure 4:**
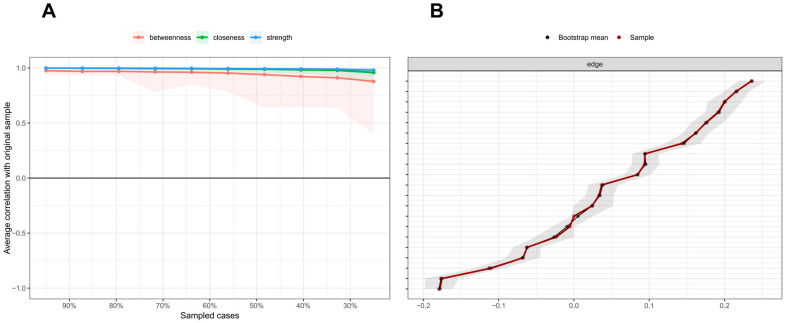
Analysis of network stability and edge-weight accuracy. (**A**) Centrality stability analysis for the overall older adult population; (**B**) Edge-weight accuracy analysis for the overall older adult population. (In panel (**A**), the curve represents the correlation between centrality measures re-estimated after case exclusion via bootstrap and the original sample centrality measures; in panel (**B**), the gray area indicates the 95% confidence interval estimated by non-parametric bootstrap, the red line represents the original sample edge-weight estimates, and black dots represent bootstrap means).

In the overall older adult population, stability and accuracy analyses were conducted on the network models. The results of case-dropping bootstrap tests are shown in Figure 4A. The CS coefficients for strength, closeness centrality, and betweenness centrality were all 0.75, indicating that the ranking of centrality measures remained highly consistent between the original and re-estimated networks even when the sample size was reduced by up to 75%, suggesting good stability of the network centrality indicators. The correlation curves for strength and closeness centrality remained close to 1 throughout, demonstrating high stability; in contrast, betweenness centrality showed a slight decline as the proportion of removed samples increased, yet maintained overall robust stability. Further assessment of edge-weight accuracy using non-parametric bootstrap methods is shown in Figure 4B. Results indicated strong consistency between bootstrap means and original sample estimates, with most edge weights exhibiting narrow 95% confidence intervals, suggesting high accuracy and reliability in edge-weight estimation and an overall stable network structure. Stability test results for male and female older adults were similar to those of the overall older adult population (see Supplementary Figures S4A,B and S5A,B).

### 3.6. Sensitivity Analysis

To evaluate whether our network findings were sensitive to the number of factors extracted, we repeated the network estimation using 3-factor and 5-factor solutions. In the 3-factor solution (factor loadings shown in Supplementary Table S14), FD-1 (vegetable-oil–legumes–eggs–milk pattern), FD-2 (fruits–vegetables–fish pattern), and FD-3 (garlic–nuts–salt-preserved vegetables pattern) were identified. FD-1 remained the node with the highest strength centrality (strength = 0.901), followed by FD-2 (strength = 0.695) and FD-3 (strength = 0.652) (Supplementary Table S15; Supplementary Figures S6–S8). In the 5-factor solution (factor loadings shown in Supplementary Table S16), FD-1 (legumes–fish–eggs pattern), FD-2 (fruits–vegetables pattern), FD-3 (garlic–nuts–salt-preserved vegetables pattern), FD-4 (meat pattern), and FD-5 (milk pattern) were identified. FD-1 remained the node with the highest strength centrality (strength = 0.954), followed by FD-2 (strength = 0.786) and FD-5 (strength = 0.729). (FD-5 (milk pattern) was a component of FD-1 (legumes–eggs–milk pattern) in the four-factor solution (Supplementary Table S17; Supplementary Figures S9–S11). Across all three solutions (3-factor, 4-factor, and 5-factor), FD-1 (legumes–eggs–milk pattern) consistently ranked as the top node in strength centrality, and FD-2 (fruits–vegetables pattern) consistently ranked among the top two, indicating that the centrality rankings of the key dietary pattern nodes were not highly sensitive to the number of factors retained. The CS coefficients for all centrality measures (strength, closeness centrality, and betweenness centrality) were consistently 0.75 across both the 3-factor and 5-factor solutions. In addition, most edge weights exhibited narrow 95% confidence intervals across both solutions, indicating high accuracy and reliability in edge-weight estimation, thereby suggesting good network stability.

## 4. Discussion

Based on a nationally representative sample of older adults, this study systematically explored the associative structure between food intake patterns: FD-1 (legumes–eggs–milk pattern), FD-2 (fruits–vegetables pattern), FD-3 (garlic–nuts–salt-preserved vegetables pattern) and FD-4 (meat–fish pattern), and each dimension of frailty among Chinese older adults using network analysis. FD-1 (legumes–eggs–milk pattern) and FD-2 (fruits–vegetables pattern) were identified as prominent food intake pattern nodes in the whole network, indicating that frailty in older adults may be associated not only with single foods but also with key food combinations in the overall dietary structure. Notably, FD-1 showed the highest centrality, suggesting this dietary pattern may be linked to better outcomes of frailty by supporting nutritional status, muscle function, and physical reserves. FD-2 appears more closely related to BS (basic status), including subjective health perception, emotional well-being, and general health condition. Furthermore, BS (basic status) and BA (basic abilities) played pivotal roles in the frailty dimensions, particularly BA, which exhibited the highest bridge strength both in the overall network and within the frailty subnetwork. Overall, this study identifies FD-1, FD-2, BS, and BA as nodes with high centrality in the network linking diet and frailty, providing new evidence for early identification, dietary factors potentially associated with frailty, and functional maintenance in older adults.

FD-1 (legumes–eggs–milk pattern) is characterized by a high-nutrient-density dietary combination centered on legumes, eggs, and milk, providing high-quality protein, vitamin B12, and various essential micronutrients, and may be associated with vitamin D nutritional status. Protein intake [47,48], vitamin D [49,50,51], and vitamin B12 status [52,53] have each been associated with frailty risk, potentially through pathways involving muscle maintenance (protein), neuromuscular and bone function (vitamin D), and neurological function (B12) [51,53,54,55,56,57]. FD-2 (fruits–vegetables pattern) is identified by a plant-based dietary combination centered on fruits and vegetables and is rich in dietary fiber, vitamin C, carotenoids, flavonols, flavonoids, and various phytochemicals. Higher intakes of fruits and vegetables, carotenoids, flavonols, and flavonoids have been associated with lower frailty risk, potentially through dietary fiber-mediated gut microbiota modulation and inflammation reduction [58,59,60,61,62,63,64,65,66], as well as antioxidant and anti-inflammatory phytochemicals that support muscle function [64,67,68]. This study identifies BA (basic abilities) and BS (basic status) as nodes with high centrality within the frailty network, with BA exhibiting the highest bridge strength, suggesting it may serve as a notable bridging node within the network. However, bridge centrality is a mathematical property of the network structure rather than evidence of a causal mechanism, and this interpretation should be considered hypothesis-generating rather than conclusive. BA encompasses fundamental daily living skills, including bathing, dressing, toileting, transferring, bladder and bowel control, and eating. As core components of activities of daily living (ADLs), impairments in these skills directly reflect declines in self-care capacity and physical function. Higher dietary diversity has been associated with lower ADL disability risk [69], while ADL/IADL impairments are linked to increased frailty risk [70]; sustained dietary diversity is also associated with reduced frailty risk [71,72]. Thus, the impact of food intake may manifest as changes in basic daily living abilities, positioning BA as a potential bridge linking diet to frailty progression. Similar to Zheng et al. [24], BA may play a bridging role connecting food intake, physical function, and frailty in this network. However, as with all network-derived metrics, this interpretation should be viewed as hypothesis-generating. BS also demonstrates high centrality, indicating that subjective health dimensions—including health status assessment, emotional experience, positive psychology, and autonomous decision-making—also contribute to the formation of the frailty network. Existing systematic reviews and clinical practice guidelines suggest that nutritional interventions, protein supplementation, dietary optimization, and combined nutrition–physical activity interventions can improve frailty and physical function [73,74]. In this context, BA may represent a priority node of interest for identifying factors potentially associated with diet-related frailty.

The sex-stratified analysis of this study revealed that in the female networks, FD-2 (fruits–vegetables pattern) was more closely associated with FD-4 (meat–fish pattern), suggesting a greater tendency for plant-based foods and high-quality animal protein intake to co-occur. This may be related to sex differences in dietary behaviors; previous studies have shown that men tend to prefer red meat and processed meats, while women are more inclined toward healthier choices such as vegetables, whole grains, and soy products [75,76]. Additionally, the relationship between dietary diversity and frailty may differ by sex [77], and associations between diet quality and frailty also exhibit sex-specific patterns [78]. In contrast, in the male networks, FD-1 (legumes–eggs–milk pattern) showed stronger connections with FD-3 (garlic–nuts–salt-preserved vegetables pattern), and FD-1 had a more prominent centrality, indicating that the frailty-related food network among older men may emphasize the role of protein sources and muscle function maintenance. Previous research has demonstrated that protein intake is linked to frailty outcomes [11]; meanwhile, skeletal muscle exhibits sex differences in structure, metabolism, and aging trajectories [79,80]. Therefore, the female network may reflect the overall impact of dietary diversity and food combination structures, whereas the male network may highlight the connection between high-quality protein sources and factors potentially associated with muscle function maintenance.

The strength of this study lies in its use of a large, nationally representative sample of older adults and the application of network analysis to explore the relationship between food intake patterns and various dimensions of frailty among Chinese older adults. Nevertheless, several limitations remain. First, this study is based on cross-sectional data; thus, network analysis can only reveal the association structure between dietary patterns and frailty dimensions, without establishing temporal sequence or causal relationships. We acknowledge that reverse causation is a plausible alternative explanation for the observed associations, particularly given the advanced age of our study population (mean age 85.3 years). It is possible that frailty-related declines in physical function, appetite, or the ability to prepare and purchase food lead to changes in dietary patterns, rather than dietary patterns influencing frailty. Our network analysis captures conditional associations at a single time point and cannot distinguish between these directional possibilities. Future longitudinal studies are needed to clarify the predictive roles and potential causal pathways of FD-1, FD-2, and BA in the onset and progression of frailty. Second, food intake data were self-reported, which may introduce recall bias and reporting bias. In addition, the heterogeneous scoring system of the FFQ, with both binary and ordinal variables across food groups, may introduce measurement variance inconsistency, which could affect comparability and the estimation of dietary pattern structures. Furthermore, dietary supplement use and medication use were not included as covariates due to data limitations, which may introduce residual confounding. Additionally, due to data availability constraints, information on specific intake amounts, cooking methods, processing techniques, and food quality was not included. Future research should employ more detailed dietary assessment tools to better distinguish different food subcategories and processing forms, such as dark versus light-colored vegetables, fresh versus salted vegetables, fish versus red meat or processed meats, and full-fat versus low-fat dairy products. Third, although this study used 35 health deficit indicators to construct the frailty index, providing a relatively comprehensive reflection of multidimensional health status in older adults, objective biomarkers such as inflammatory markers and hormone levels were not included, limiting the understanding of underlying biological mechanisms linking diet and frailty. Future studies could integrate biomarker data with multilevel network analysis to further elucidate the complex interplay among food intake patterns, functional status, and frailty progression.

## 5. Conclusions

In summary, this study reveals, through network analysis, the complex associations between dietary patterns and multidimensional frailty in older adults. FD-1 (legumes–eggs–milk pattern), FD-2 (fruits–vegetables pattern), and BA (basic ability) showed high centrality in the network, suggesting that high-quality protein sources, plant-based food consumption, and maintenance of basic functional capacity may be factors of interest associated with frailty. Our results suggest potential avenues for future longitudinal and intervention studies; however, such studies are needed to determine whether modifying these dietary patterns or functional status can reduce frailty risk.

## Figures and Tables

**Table 1:** Characteristics of study participants (*n* = 14,140).

Characteristic	Total Participants	Non-Frailty	Pre-Frailty	Frailty	*p* Value
	(*n* = 14,140)	(*n* = 3072)	(*n* = 6552)	(*n* = 4516)	
Age, years, mean (SD)	85.33 (11.57)	78.48 (9.63)	84.15 (11.02)	91.71 (10.27)	
Age group, *n* (%)					<0.001
<75 years	3037 (21.5)	1231 (40.1)	1480 (22.6)	326 (7.2)	
≥75 years	11,103 (78.5)	1841 (59.9)	5072 (77.4)	4190 (92.8)	
Sex, *n* (%)					<0.001
Male	6214 (43.9)	1618 (52.7)	2987 (45.6)	1609 (35.6)	
Female	7926 (56.1)	1454 (47.3)	3565 (54.4)	2907 (64.4)	
Residence, *n* (%)					0.065
Urban	2033 (16.8)	398 (15.7)	922 (16.6)	713 (17.8)	
Rural	10,062 (83.2)	2136 (84.3)	4642 (83.4)	3284 (82.2)	
Economic status, *n* (%)					<0.001
Affluent	2750 (19.6)	764 (25.0)	1255 (19.3)	731 (16.5)	
Moderate	9782 (69.9)	2120 (69.3)	4615 (71.0)	3047 (68.6)	
Deprived	1468 (10.5)	173 (5.7)	632 (9.7)	663 (14.9)	
Cohabitation status, *n* (%)					<0.001
Not living alone	11,739 (84.1)	2442 (80.5)	5316 (82.3)	3981 (89.0)	
Living alone	2227 (15.9)	592 (19.5)	1145 (17.7)	490 (11.0)	
Marital status, *n* (%)					<0.001
Married/Living with partner/Separated	5813 (41.5)	1844 (60.4)	2854 (44.0)	1115 (25.0)	
Divorced/Widowed/Never married	8196 (58.5)	1210 (39.6)	3637 (56.0)	3349 (75.0)	
Smoking status, *n* (%)					<0.001
Smoker	2078 (14.8)	652 (21.5)	1051 (16.2)	375 (8.4)	
Non-smoker	11,935 (85.2)	2387 (78.5)	5447 (83.8)	4101 (91.6)	
Drinking status, *n* (%)					<0.001
Drinker	1999 (14.3)	693 (22.9)	988 (15.3)	318 (7.2)	
Non-drinker	11,940 (85.7)	2338 (77.1)	5479 (84.7)	4123 (92.8)	
Physical exercise, *n* (%)					<0.001
Exerciser	4261 (30.5)	1361 (44.9)	2175 (33.6)	725 (16.3)	
Non-exerciser	9704 (69.5)	1672 (55.1)	4298 (66.4)	3734 (83.7)	
BMI, kg/m^2^, mean (SD)	22.20 (3.82)	22.69 (3.49)	22.37 (3.79)	21.49 (4.05)	
BMI category, *n* (%)					<0.001
Underweight	2166 (17.1)	335 (11.3)	960 (15.6)	871 (24.5)	
Normal	7655 (60.3)	1883 (63.5)	3762 (61.0)	2010 (56.6)	
Overweight	2528 (19.9)	681 (23.0)	1265 (20.5)	582 (16.4)	
Obese	336 (2.6)	66 (2.2)	179 (2.9)	91 (2.6)	

Note: Due to missing values for some variables, the total number of respondents may vary across variables. The number of missing values was as follows: residence (*n* = 2045), economic status (*n* = 140), cohabitation status (*n* = 174), marital status (*n* = 131), smoking status (*n* = 127), drinking status (*n* = 201), physical exercise (*n* = 175), and BMI category (*n* = 1455). Percentages were calculated based on the effective sample size.

## Data Availability

The original data presented in the study are openly available in Chinese Longitudinal Healthy Longevity Survey (CLHLS) at https://opendata.pku.edu.cn/dataverse/CHADS (accessed on 3 May 2026).

## Supplementary Materials

The following supporting information is available online: https://www.mdpi.com/article/10.3390/nu18142310/s1, Table S1: Food items and scoring criteria used in the food frequency questionnaire; Table S2: Dimensions and health-related indicators of the frailty index; Table S3: Measures of Sampling Adequacy (MSA) (4-factor solution); Figure S1: Scree plot of factor analysis (4-factor solution); Table S4: Factor loadings (4-factor solution); Table S5: Correlation matrix for the overall sample of older adults (4-factor solution); Table S6: Correlation matrix for male older adults (4-factor solution); Table S7: Correlation matrix for female older adults (4-factor solution); Figure S2A: Network structure of food intake patterns and frailty among male older adults (4-factor solution); Figure S2B: Network structure of food intake patterns and frailty among female older adults (4-factor solution); Table S8: Centrality indicators for the overall sample of older adults (4-factor solution); Table S9: Centrality indicators for male older adults (4-factor solution); Table S10: Centrality indicators for female older adults (4-factor solution); Figure S3A: Centrality indicators for the food intake patterns and frailty network among male older adults (4-factor solution); Figure S3B: Centrality indicators for the food intake patterns and frailty network among female older adults (4-factor solution); Table S11: Bridge centrality indicators for the overall sample of older adults (4-factor solution); Table S12: Bridge centrality indicators for male older adults (4-factor solution); Table S13: Bridge centrality indicators for female older adults (4-factor solution); Figure S4A: Centrality stability analysis for male older adults (4-factor solution); Figure S4B: Edge-weight accuracy analysis for male older adults (4-factor solution); Figure S5A: Centrality stability analysis for female older adults (4-factor solution); Figure S5B: Edge-weight accuracy analysis for female older adults (4-factor solution); Table S14: Factor loadings (3-factor solution); Table S15: Centrality indicators for the overall sample of older adults (3-factor solution); Figure S6: Network structure of food intake patterns and frailty among overall older adults (3-factor solution); Figure S7: Centrality indicators for the food intake patterns and frailty network among overall older adults (3-factor solution); Figure S8A: Centrality stability analysis for the overall older adult population (3-factor solution); Figure S8B: Edge-weight accuracy analysis for the overall older adult population (3-factor solution); Table S16: Factor loadings (5-factor solution); Table S17: Centrality indicators for the overall sample of older adults (5-factor solution; Figure S9: Network structure of food intake patterns and frailty among overall older adults (5-factor solution); Figure S10: Centrality indicators for the food intake patterns and frailty network among overall older adults (5-factor solution); Figure S11A: Centrality stability analysis for the overall older adult population (5-factor solution); Figure S11B: Edge-weight accuracy analysis for the overall older adult population (5-factor solution).
