# Supplementary material for: Network Analysis of Food Intake Patterns and Frailty Dimensions in Chinese Older Adults: A National Cross-Sectional Study
Source: Nutrients. 2026 Jul 14;18(14):2310. doi: 10.3390/nu18142310 (PMC13414739; doi:10.3390/nu18142310)
Supplement: Supplementary file 1 [file nutrients-18-02310-s001.zip › nutrients-4397725-supplementary.pdf]

Supplementary material

Contents

Table S1. Food items and scoring criteria used in the food frequency questionnaire. ……1
Table S2. Dimensions and health-related indicators of the frailty index. ……2
Table S3. Measures of Sampling Adequacy (MSA) (4-factor solution). ……4
Figure S1. Scree plot of factor analysis (4-factor solution). ……5
Table S4. Factor loadings (4-factor solution). ……6
Table S5. Correlation matrix for the overall sample of older adults (4-factor solution). ……7
Table S6. Correlation matrix for male older adults (4-factor solution). ……8
Table S7. Correlation matrix for female older adults (4-factor solution). ……9
Figure S2A. Network structure of food intake patterns and frailty among male older adults (4-factor solution). ……10
Figure S2B. Network structure of food intake patterns and frailty among female older adults (4-factor solution). ……10
Table S8. Centrality indicators for the overall sample of older adults (4-factor solution). ……11
Table S9. Centrality indicators for male older adults (4-factor solution). ……12
Table S10. Centrality indicators for female older adults (4-factor solution). ……13
Figure S3A. Centrality indicators for the food intake patterns and frailty network among male older adults (4-factor solution). ……14
Figure S3B. Centrality indicators for the food intake patterns and frailty network among female older adults (4-factor solution). ……14
Table S11. Bridge centrality indicators for the overall sample of older adults (4-factor solution). ……15
Table S12. Bridge centrality indicators for male older adults (4-factor solution). ……16
Table S13. Bridge centrality indicators for female older adults (4-factor solution). ……17
Figure S4A. Centrality stability analysis for male older adults (4-factor solution). ……18
Figure S4B. Edge-weight accuracy analysis for male older adults (4-factor solution). ……18
Figure S5A. Centrality stability analysis for female older adults (4-factor solution). ……19

Figure S5B. Edge-weight accuracy analysis for female older adults (4-factor solution). 19
Table S14. Factor loadings (3-factor solution). 20
Table S15. Centrality indicators for the overall sample of older adults (3-factor solution). 21
Figure S6. Network structure of food intake patterns and frailty among overall older adults (3-factor solution). 22
Figure S7. Centrality indicators for the food intake patterns and frailty network among overall older adults (3-factor solution). 23
Figure S8A. Centrality stability analysis for the overall older adult population (3-factor solution). 24
Figure S8B. Edge-weight accuracy analysis for the overall older adult population (3-factor solution). 24
Table S16. Factor loadings (5-factor solution). 25
Table S17. Centrality indicators for the overall sample of older adults (5-factor solution). 26
Figure S9. Network structure of food intake patterns and frailty among overall older adults (5-factor solution). 27
Figure S10. Centrality indicators for the food intake patterns and frailty network among overall older adults (5-factor solution). 28
Figure S11A. Centrality stability analysis for the overall older adult population (5-factor solution). 29
Figure S11B. Edge-weight accuracy analysis for the overall older adult population (5-factor solution). 29

Table S1. Food items and scoring criteria used in the food frequency questionnaire.

No.	Variables	Data type	Score assignment
1	Whole grains	Binary	Yes = 1; no = 0
2	Vegetable oil	Binary	Yes = 1; no = 0
3	Frequency of fresh fruits intake	Ordinal	Almost every day = 4; except in winter = 3; occasionally = 2; rarely or never = 1
4	Frequency of fresh vegetables intake	Ordinal	Almost every day = 4; except in winter = 3; occasionally = 2; rarely or never = 1
5	Frequency of legumes intake	Ordinal	Almost every day = 5; ≥1 time/week = 4; ≥1 time/month = 3; occasionally = 2; rarely or never = 1
6	Frequency of garlic intake	Ordinal	Almost every day = 5; ≥1 time/week = 4; ≥1 time/month = 3; occasionally = 2; rarely or never = 1
7	Frequency of nut products intake	Ordinal	Almost every day = 5; ≥1 time/week = 4; ≥1 time/month = 3; occasionally = 2; rarely or never = 1
8	Frequency of tea consumption	Ordinal	Almost every day = 5; ≥1 time/week = 4; ≥1 time/month = 3; occasionally = 2; rarely or never = 1
9	Frequency of salt-preserved vegetables intake	Ordinal	Almost every day = 5; ≥1 time/week = 4; ≥1 time/month = 3; occasionally = 2; rarely or never = 1
10	Frequency of sugar intake	Ordinal	Almost every day = 5; ≥1 time/week = 4; ≥1 time/month = 3; occasionally = 2; rarely or never = 1
11	Frequency of meat intake	Ordinal	Almost every day = 5; ≥1 time/week = 4; ≥1 time/month = 3; occasionally = 2; rarely or never = 1
12	Frequency of fish intake	Ordinal	Almost every day = 5; ≥1 time/week = 4; ≥1 time/month = 3; occasionally = 2; rarely or never = 1
13	Frequency of eggs intake	Ordinal	Almost every day = 5; ≥1 time/week = 4; ≥1 time/month = 3; occasionally = 2; rarely or never = 1
14	Frequency of milk products intake	Ordinal	Almost every day = 5; ≥1 time/week = 4; ≥1 time/month = 3; occasionally = 2; rarely or never = 1

Note: Responses coded as “don’t know” or “missing” in the codebook were treated as missing values.

Table S2. Dimensions and health-related indicators of the frailty index.

No.	Dimensions	Variables	Data type	Score assignment
1	Basic status	Self-reported health	Ordinal	Very good = 0; good = 0.25; so so = 0.5; bad = 0.75; very bad = 1
2		Are you nervous and scared?	Ordinal	Always = 1; often = 0.75; sometimes = 0.5; seldom = 0.25; never = 0
3		Do you think that the older you are, the less useful you are, and the hard work?	Ordinal	Always = 1; often = 0.75; sometimes = 0.5; seldom = 0.25; never = 0
4		Look on the bright side of things	Ordinal	Always = 0; often = 0.25; sometimes = 0.5; seldom = 0.75; never = 1
5		Do you like to keep things clean and tidy?	Ordinal	Always = 0; often = 0.25; sometimes = 0.5; seldom = 0.75; never = 1
6		Do you have your own business?	Ordinal	Always = 0; often = 0.25; sometimes = 0.5; seldom = 0.75; never = 1
7		The health of interviewee rated by interviewer	Ordinal	Surprisingly healthy = 0; relatively healthy = 0.33; moderately ill = 0.67; very ill = 1
8	Basic abilities	Bathing	Ordinal	Without assistance = 0; one part assistance = 0.5; more than one part assistance = 1
9		Dressing	Ordinal	Without assistance = 0; one part assistance = 0.5; more than one part assistance = 1
10		Toileting	Ordinal	Without assistance = 0; one part assistance = 0.5; more than one part assistance = 1
11		Transferring	Ordinal	Without assistance = 0; one part assistance = 0.5; more than one part assistance = 1
12		Continence	Ordinal	Without assistance = 0; one part assistance = 0.5; more than one part assistance = 1
13		Feeding	Ordinal	Without assistance = 0; one part assistance = 0.5; more than one part assistance = 1
14		Do you do house work at present?	Ordinal	Almost everyday = 0; not daily, but once for a week = 0.25; not weekly, but at least once for a month = 0.5; not monthly, but sometimes = 0.75; never = 1
15		Visual function: can you see the break in the circle?	Ordinal	Can see and distinguish = 0; can see only = 0.33; can't see = 0.67; blind = 1
16		Do you have any difficulty with your hearing?	Ordinal	Yes, without hearing aid = 0; yes, but needs hearing aid = 0.33; partly, despite hearing aid = 0.67; no = 1
17		Heart rhythm	Binary	$< 80=0$; $\geq 80=1$
18		Hand behind neck	Ordinal	Both hands=0; right hand=0.5; left hand=0.5; neither hand=1
19		Hand behind lower back	Ordinal	Both hands=0; right hand=0.5; left hand=0.5; neither hand=1
20		Able to stand up from sitting in a chair?	Ordinal	Yes, without using hands=0; yes, using hands=0.5; no=1

21		Able to pick up a book from the floor?	Ordinal	Yes, standing=0; yes, sitting=0.5; no=1
22		Able to use chopsticks to eat?	Binary	Yes=0; no=1
23		Steps used to turn around 360 with help?	Ordinal	≤4 = 0; >4 = 0.5; cannot turn around = 1
24	Medical history	Number of times suffering from serious illness within the past two years	Ordinal	0 = 0; 1 = 1; >1 = 2
25		Suffering from hypertension?	Binary	Yes = 1; no = 0
26		Suffering from heart disease?	Binary	Yes = 1; no = 0
27		Suffering from diabetes?	Binary	Yes = 1; no = 0
28		Suffering from stroke or cvd?	Binary	Yes = 1; no = 0
29		Suffering from bronchitis; emphysema; pneumonia; asthma?	Binary	Yes = 1; no = 0
30		Suffering from tuberculosis?	Binary	Yes = 1; no = 0
31		Suffering from cancer?	Binary	Yes = 1; no = 0
32		Suffering from gastric or duodenal ulcer?	Binary	Yes = 1; no = 0
33		Suffering from parkinson's disease?	Binary	Yes = 1; no = 0
34		Suffering from bedsore?	Binary	Yes = 1; no = 0
35		Suffering from dementia?	Binary	Yes = 1; no = 0

Note: Responses coded as “don’t know” or “missing” in the codebook were treated as missing values.

Table S3. Measures of Sampling Adequacy (MSA) (4-factor solution).

No.	Food items	MSA
1	Whole grain	0.62
2	Vegetable oil	0.64
3	Fruits	0.79
4	Vegetables	0.71
5	Legumes	0.80
6	Garlic	0.80
7	Nuts	0.81
8	Tea	0.82
9	Salt-preserved vegetables	0.67
10	Sugar	0.76
11	Meat	0.68
12	Fish	0.76
13	Eggs	0.79
14	Milk	0.77

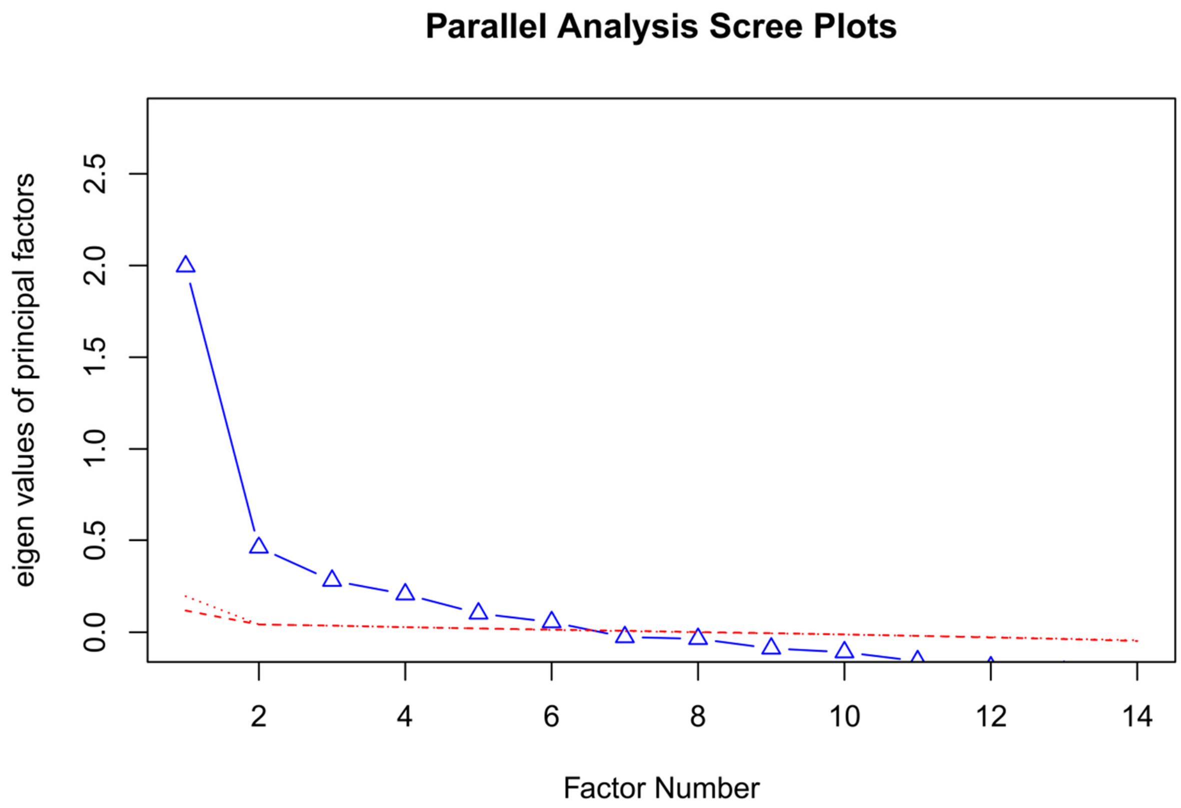

Figure S1. Scree plot of factor analysis (4-factor solution).

Table S4. Factor loadings (4-factor solution).

Food items	FD-1 (legumes – eggs – milk pattern)	FD-2 (fruits – vegetables pattern)	FD-3 (garlic – nuts – salt-preserved vegetables pattern)	FD-4 (meat – fish pattern)
Whole grain				
Vegetable oil				
Fruits		0.58		
Vegetables		0.44		
Legumes	0.39			
Garlic			0.40	
Nuts			0.37	
Tea				
Salt-preserved vegetables			0.45	
Sugar				
Meat				0.79
Fish				0.32
Eggs	0.62			
Milk	0.51			

Note: Factor loadings < 0.30 are not shown. Whole grains, vegetable oil, tea, and sugar did not load substantially on any factor (all loadings < 0.30) and were therefore not assigned to any dietary pattern in the 4-factor solution. Loadings ⩾ 0.30 were considered meaningful for pattern interpretation.

Table S5. Correlation matrix for the overall sample of older adults (4-factor solution).

Node	BS	BA	MH	FD-1	FD-2	FD-3	FD-4
BS	1	0.241	0.166	-0.165	-0.253	-0.172	-0.102
BA	0.241	1	0.067	0.047	-0.131	-0.195	-0.067
MH	0.166	0.067	1	0.100	0.031	0.044	0.015
FD-1	-0.165	0.047	0.100	1	0.283	0.304	0.291
FD-2	-0.253	-0.131	0.031	0.283	1	0.224	0.304
FD-3	-0.172	-0.195	0.044	0.304	0.224	1	0.212
FD-4	-0.102	-0.067	0.015	0.291	0.304	0.212	1

Note: BS, basic status; BA, basic ability; MH, medical history; FD-1, legumes–eggs–milk pattern; FD-2, fruits–vegetables pattern; FD-3, garlic–nuts–salt-preserved vegetables pattern; FD-4, meat–fish pattern.

Table S6. Correlation matrix for male older adults (4-factor solution).

Node	BS	BA	MH	FD-1	FD-2	FD-3	FD-4
BS	1	0.271	0.188	-0.152	-0.260	-0.182	-0.123
BA	0.271	1	0.122	0.028	-0.154	-0.181	-0.108
MH	0.188	0.122	1	0.097	0.029	0.027	-0.002
FD-1	-0.152	0.028	0.097	1	0.296	0.304	0.270
FD-2	-0.260	-0.154	0.029	0.296	1	0.214	0.287
FD-3	-0.182	-0.181	0.027	0.304	0.214	1	0.191
FD-4	-0.123	-0.108	-0.002	0.270	0.287	0.191	1

Note: BS, basic status; BA, basic ability; MH, medical history; FD-1, legumes–eggs–milk pattern; FD-2, fruits–vegetables pattern; FD-3, garlic–nuts–salt-preserved vegetables pattern; FD-4, meat–fish pattern.

Table S7. Correlation matrix for female older adults (4-factor solution).

Node	BS	BA	MH	FD-1	FD-2	FD-3	FD-4
BS	1	0.215	0.148	-0.166	-0.250	-0.157	-0.073
BA	0.215	1	0.033	0.072	-0.119	-0.195	-0.027
MH	0.148	0.033	1	0.102	0.033	0.056	0.026
FD-1	-0.166	0.072	0.102	1	0.272	0.298	0.299
FD-2	-0.250	-0.119	0.033	0.272	1	0.231	0.315
FD-3	-0.157	-0.195	0.056	0.298	0.231	1	0.216
FD-4	-0.073	-0.027	0.026	0.299	0.315	0.216	1

Note: BS, basic status; BA, basic ability; MH, medical history; FD-1, legumes–eggs–milk pattern; FD-2, fruits–vegetables pattern; FD-3, garlic–nuts–salt-preserved vegetables pattern; FD-4, meat–fish pattern.

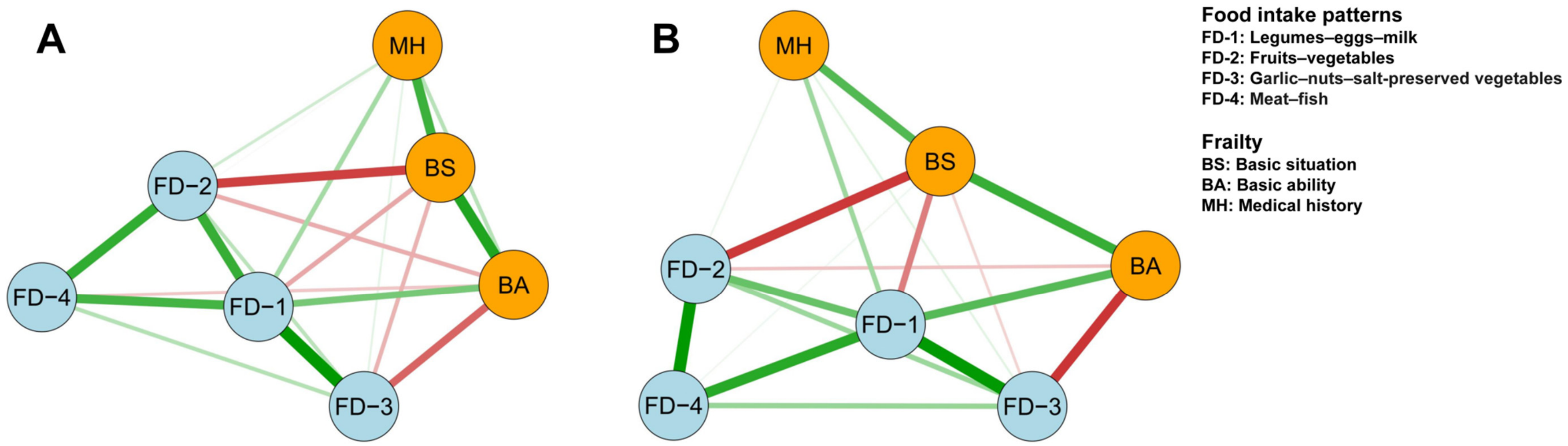

Figure S2. Network structure of food intake patterns and frailty among male and female older adults (4-factor solution). **A:** Network structure among male older adults. **B:** Network structure among female older adults. Nodes represent food intake patterns or frailty dimensions; edges indicate partial associations after controlling for other nodes. Green edges represent positive correlations; red edges represent negative correlations; edge thickness indicates the strength of association. The network layout was generated using the Fruchterman–Reingold force-directed algorithm.

Table S8. Centrality indicators for the overall sample of older adults (4-factor solution).

Node	Strength	Closeness	Betweenness
BS	0.725	0.022	6
BA	0.634	0.020	2
MH	0.368	0.014	0
FD-1	0.936	0.024	10
FD-2	0.744	0.020	2
FD-3	0.685	0.018	0
FD-4	0.530	0.017	0

Note: BS, basic status; BA, basic ability; MH, medical history; FD-1, legumes–eggs–milk pattern; FD-2, fruits–vegetables pattern; FD-3, garlic–nuts–salt-preserved vegetables pattern; FD-4, meat–fish pattern.

Table S9. Centrality indicators for male older adults (4-factor solution).

Node	Strength	Closeness	Betweenness
BS	0.757	0.021	8
BA	0.716	0.019	2
MH	0.453	0.015	0
FD-1	0.924	0.023	8
FD-2	0.777	0.022	6
FD-3	0.651	0.018	0
FD-4	0.535	0.017	0

Note: BS, basic status; BA, basic ability; MH, medical history; FD-1, legumes–eggs–milk pattern; FD-2, fruits–vegetables pattern; FD-3, garlic–nuts–salt-preserved vegetables pattern; FD-4, meat–fish pattern.

Table S10. Centrality indicators for female older adults (4-factor solution).

Node	Strength	Closeness	Betweenness
BS	0.722	0.022	6
BA	0.592	0.020	2
MH	0.316	0.014	0
FD-1	0.944	0.024	8
FD-2	0.745	0.020	2
FD-3	0.696	0.018	0
FD-4	0.554	0.018	0

Note: BS, basic status; BA, basic ability; MH, medical history; FD-1, legumes–eggs–milk pattern; FD-2, fruits–vegetables pattern; FD-3, garlic–nuts–salt-preserved vegetables pattern; FD-4, meat–fish pattern.

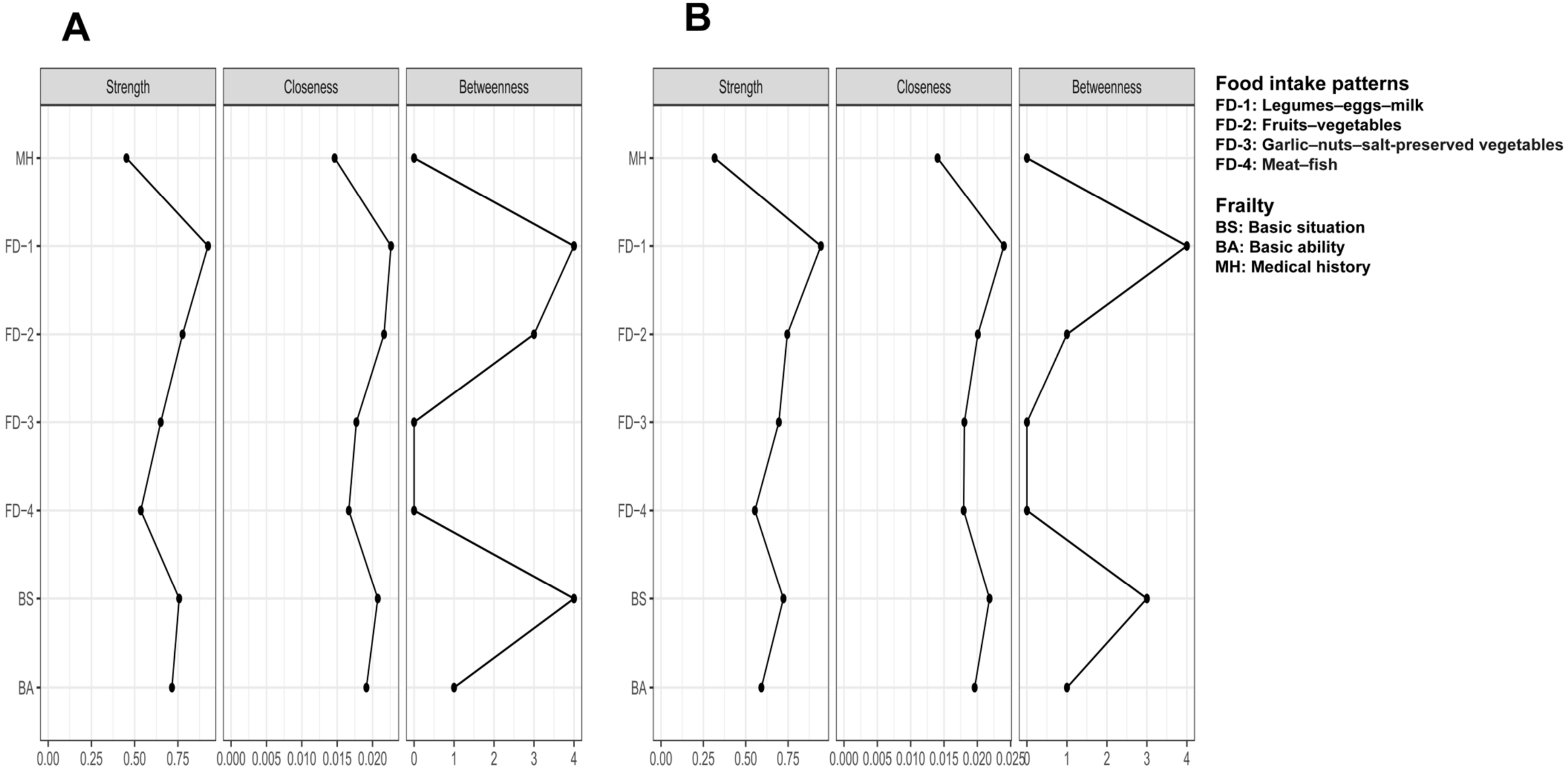

Figure S3. Centrality indicators for the food intake patterns and frailty network among male and female older adults (4-factor solution). A: Centrality indicators for male older adults. B: Centrality indicators for female older adults.

Table S11. Bridge centrality indicators for the overall sample of older adults (4-factor solution).

Node	Bridge Strength	Bridge Closeness	Bridge Betweenness
BS	0.350	0.063	0
BA	0.410	0.092	4
MH	0.169	0.069	0
FD-1	0.347	0.101	9
FD-2	0.282	0.062	0
FD-3	0.271	0.071	0
FD-4	0.029	0.066	0

Note: BS, basic status; BA, basic ability; MH, medical history; FD-1, legumes–eggs–milk pattern; FD-2, fruits–vegetables pattern; FD-3, garlic–nuts–salt-preserved vegetables pattern; FD-4, meat–fish pattern.

Table S12. Bridge centrality indicators for male older adults (4-factor solution).

Node	Bridge Strength	Bridge Closeness	Bridge Betweenness
BS	0.359	0.071	0
BA	0.395	0.092	4
MH	0.197	0.069	0
FD-1	0.334	0.107	9
FD-2	0.313	0.069	0
FD-3	0.227	0.074	0
FD-4	0.078	0.066	0

Note: BS, basic status; BA, basic ability; MH, medical history; FD-1, legumes–eggs–milk pattern; FD-2, fruits–vegetables pattern; FD-3, garlic–nuts–salt-preserved vegetables pattern; FD-4, meat–fish pattern.

Table S13. Bridge centrality indicators for female older adults (4-factor solution).

Node	Bridge Strength	Bridge Closeness	Bridge Betweenness
BS	0.377	0.063	0
BA	0.407	0.096	4
MH	0.156	0.067	0
FD-1	0.373	0.104	9
FD-2	0.275	0.060	0
FD-3	0.269	0.072	0
FD-4	0.024	0.068	0

Note: BS, basic status; BA, basic ability; MH, medical history; FD-1, legumes–eggs–milk pattern; FD-2, fruits–vegetables pattern; FD-3, garlic–nuts–salt-preserved vegetables pattern; FD-4, meat–fish pattern.

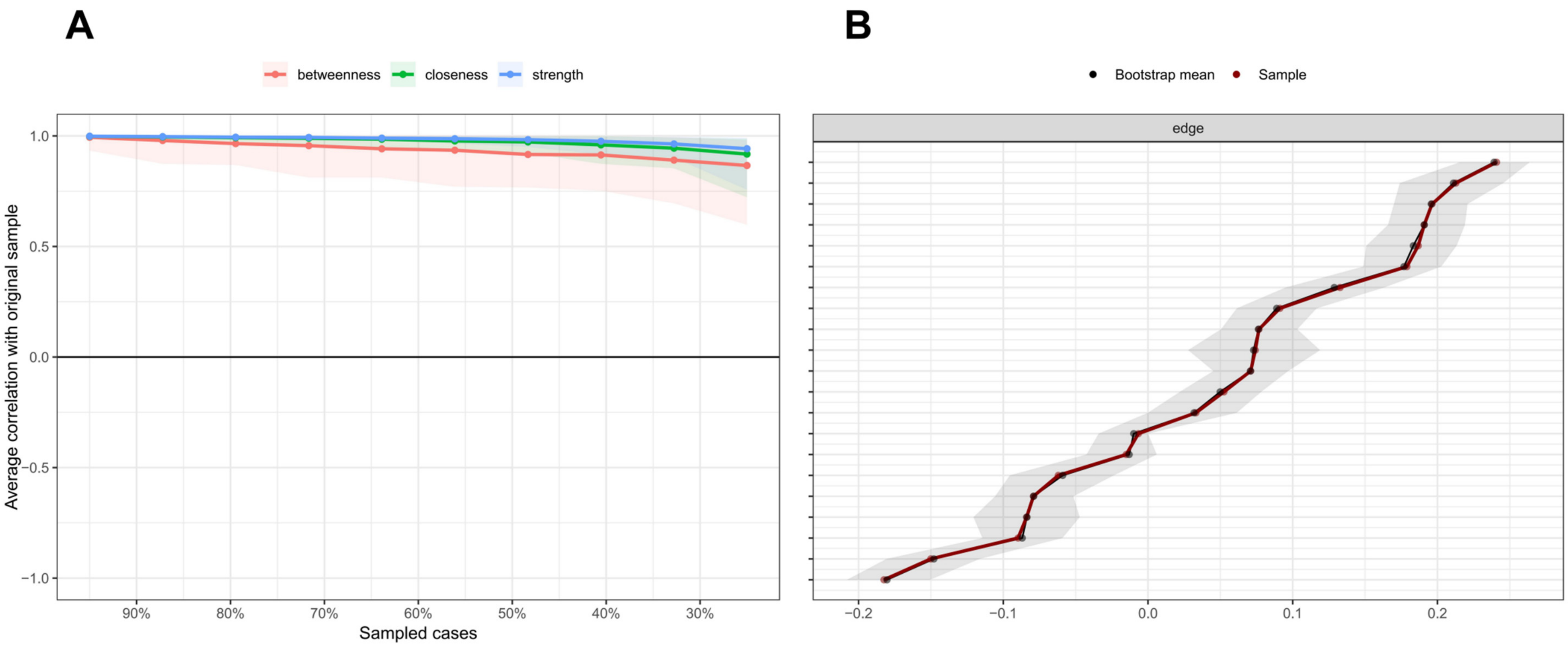

Figure S4. Analysis of network stability and edge-weight accuracy (4-factor solution). **A:** Centrality stability analysis for male older adults. **B:** Edge-weight accuracy analysis for male older adults. (In panel A, the curve represents the correlation between centrality measures re-estimated after case exclusion via bootstrap and the original sample centrality measures; in panel B, the gray area indicates the 95% confidence interval estimated by non-parametric bootstrap; the red line represents the original sample edge weight estimates; and black dots represent bootstrap means.)

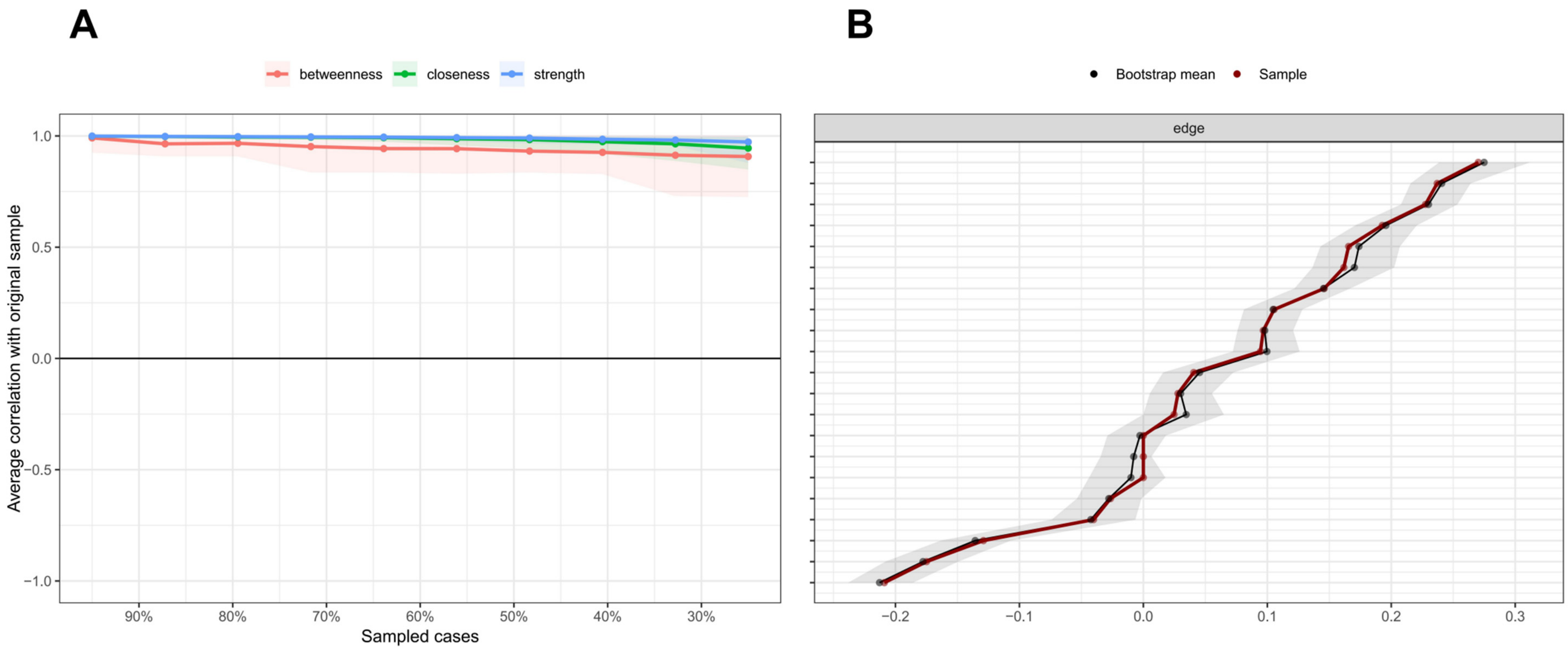

Figure S5. Analysis of network stability and edge-weight accuracy (4-factor solution). **A:** Centrality stability analysis for female older adults. **B:** Edge-weight accuracy analysis for female older adults. (In panel A, the curve represents the correlation between centrality measures re-estimated after case exclusion via bootstrap and the original sample centrality measures; in panel B, the gray area indicates the 95% confidence interval estimated by non-parametric bootstrap; the red line represents the original sample edge weight estimates; and black dots represent bootstrap means.)

Table S14. Factor loadings (3-factor solution).

Food items	FD-1 (vegetable-oil－legumes－eggs－milk pattern)	FD-2 (fruits－vegetables－fish pattern)	FD-3 (garlic－nuts－salt-preserved vegetables pattern)
Whole grain			
Vegetable oil	0.33		
Fruits		0.35	
Vegetables		0.32	
Legumes	0.34		
Garlic			0.37
Nuts			0.35
Tea			
Salt-preserved vegetables			0.50
Sugar			
Meat			
Fish		0.51	
Eggs	0.51		
Milk	0.56		

Note: Factor loadings < 0.30 are not shown. Whole grains, vegetable oil, tea, and sugar did not load substantially on any factor (all loadings < 0.30) and were therefore not assigned to any dietary pattern in the 5-factor solution. Loadings ⩾ 0.30 were considered meaningful for pattern interpretation.

Table S15. Centrality indicators for the overall sample of older adults (3-factor solution).

Node	Strength	Closeness	Betweenness
BS	0.703	0.027	4
BA	0.646	0.027	2
MH	0.370	0.019	0
FD-1	0.901	0.031	4
FD-2	0.695	0.025	0
FD-3	0.652	0.024	0

Note: BS, basic status; BA, basic ability; MH, medical history; FD-1,vegetable-oil–legumes–eggs–milk pattern; FD-2, fruits–vegetables–fish pattern; FD-3, garlic–nuts–salt-preserved vegetables pattern.

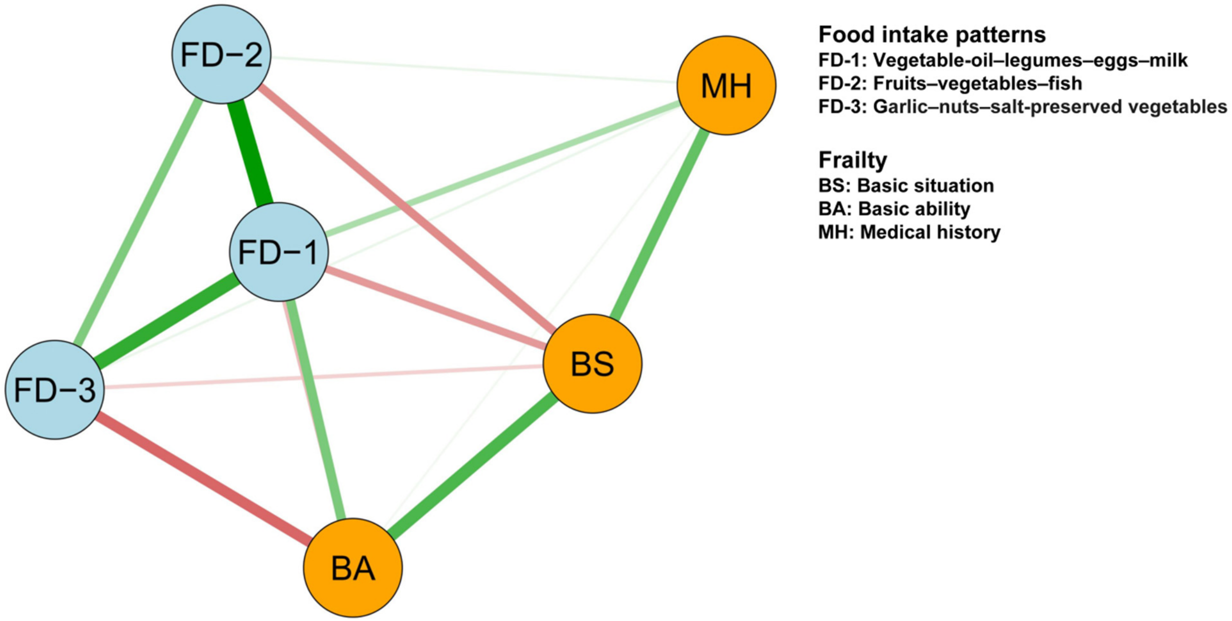

Figure S6. Network structure of food intake patterns and frailty among overall older adults (3-factor solution). Nodes represent food intake patterns or frailty dimensions; edges indicate partial associations after controlling for other nodes. Green edges represent positive correlations, red edges represent negative correlations; edge thickness indicates the strength of association.The network layout was generated using the Fruchterman–Reingold force-directed algorithm. (3-factor solution). The network layout was generated using the Fruchterman–Reingold force-directed algorithm.

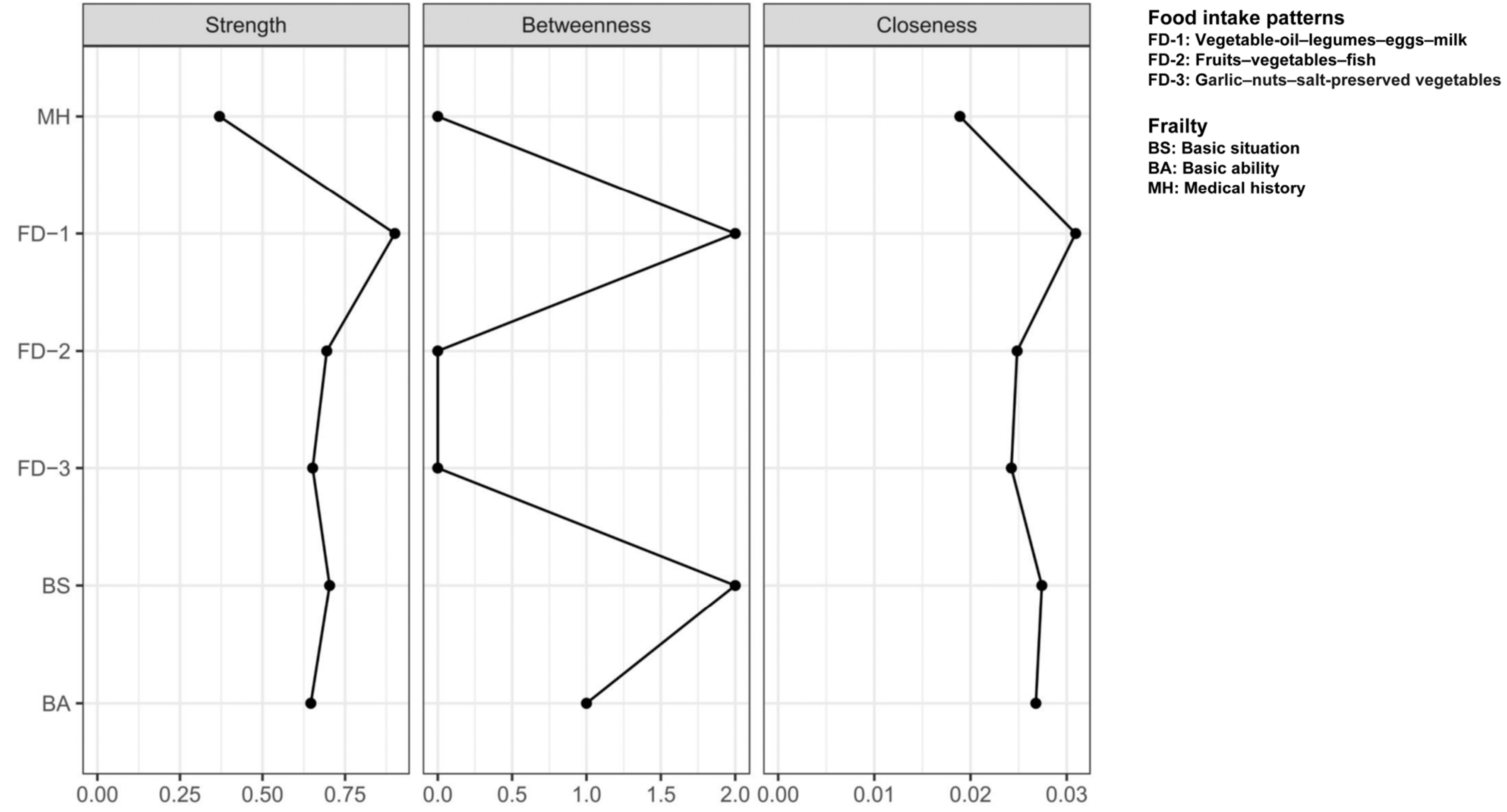

Figure S7. Centrality indicators for the food intake patterns and frailty network among overall older adults (3-factor solution).

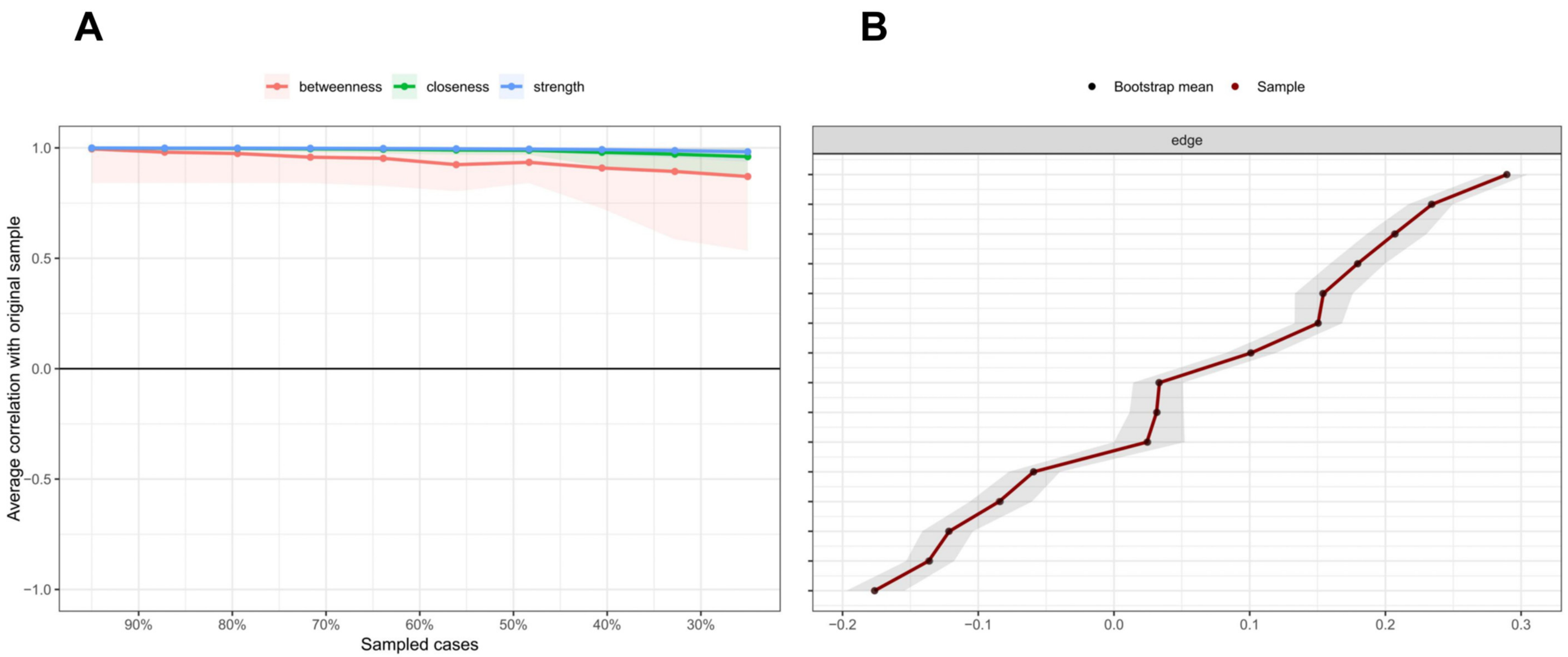

Figure S8. Analysis of network stability and edge-weight accuracy (3-factor solution). A: Centrality stability analysis for the overall older adult population; B: Edge-weight accuracy analysis for the overall older adult population. (In panel A, the curve represents the correlation between centrality measures re-estimated after case exclusion via bootstrap and the original sample centrality measures; in panel B, the gray area indicates the 95% confidence interval estimated by non-parametric bootstrap, the red line represents the original sample edge weight estimates, and black dots represent bootstrap means.) (3-factor solution).

Table S16. Factor loadings (5-factor solution).

Food items	FD-1 (legumes – fish – eggs pattern)	FD-2 (fruits – vegetables pattern)	FD-3 (garlic – nuts – salt-preserved vegetables pattern)	FD-4 (meat pattern)	FD-5 (milk pattern)
Whole grain					
Vegetable oil					
Fruits		0.57			
Vegetables		0.46			
Legumes	0.51				
Garlic			0.41		
Nuts			0.43		
Tea					
Salt-preserved vegetables			0.41		
Sugar					
Meat				0.74	
Fish	0.36				
Eggs	0.50				
Milk					0.64

Note: Factor loadings < 0.30 are not shown. Whole grains, vegetable oil, tea, and sugar did not load substantially on any factor (all loadings < 0.30) and were therefore not assigned to any dietary pattern in the 5-factor solution. Loadings ⩾ 0.30 were considered meaningful for pattern interpretation.

Table S17. Centrality indicators for the overall sample of older adults (5-factor solution).

Node	Strength	Closeness	Betweenness
BS	0.740	0.016	6
BA	0.608	0.015	2
MH	0.430	0.013	0
FD-1	0.954	0.019	14
FD-2	0.786	0.017	4
FD-3	0.678	0.015	4
FD-4	0.424	0.014	0
FD-5	0.729	0.017	6

Note: BS, basic status; BA, basic ability; MH, medical history; FD-1,legumes–fish–eggs pattern; FD-2, fruits–vegetables pattern; FD-3, garlic–nuts–salt-preserved vegetables pattern; FD-4, meat pattern; FD-5, milk pattern.

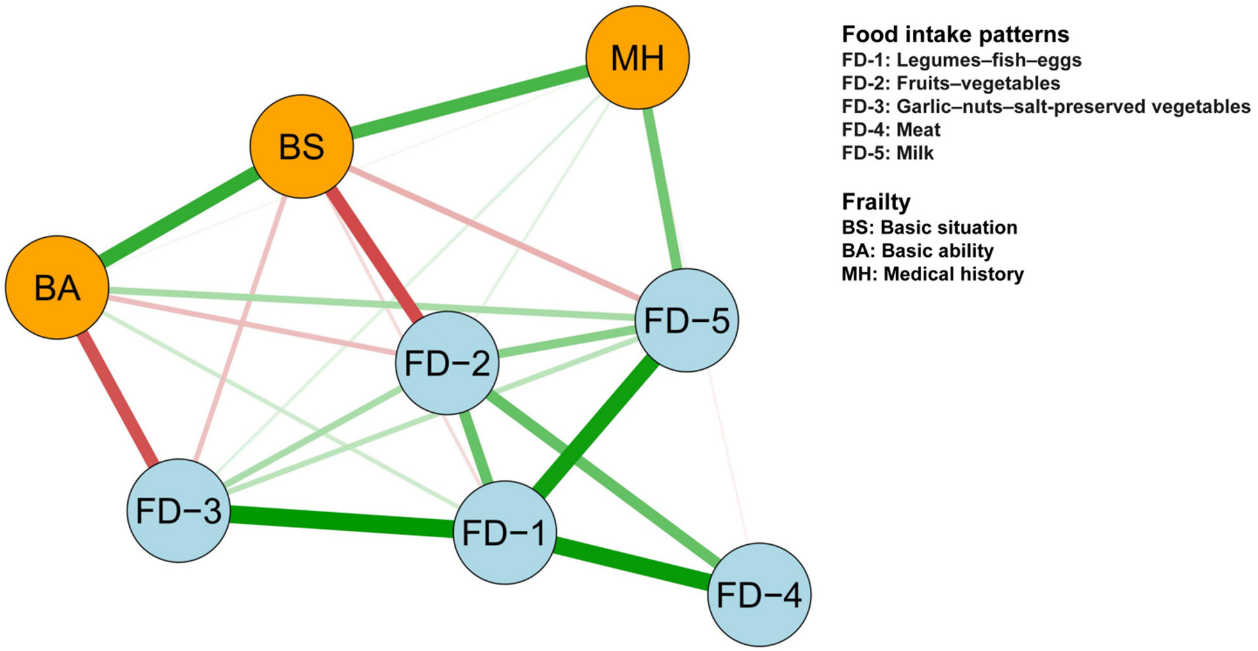

Figure S9. Network structure of food intake patterns and frailty among overall older adults (5-factor solution). Nodes represent food intake patterns or frailty dimensions; edges indicate partial associations after controlling for other nodes. Green edges represent positive correlations, red edges represent negative correlations; edge thickness indicates the strength of association.The network layout was generated using the Fruchterman – Reingold force-directed algorithm (5-factor solution). The network layout was generated using the Fruchterman – Reingold force-directed algorithm.

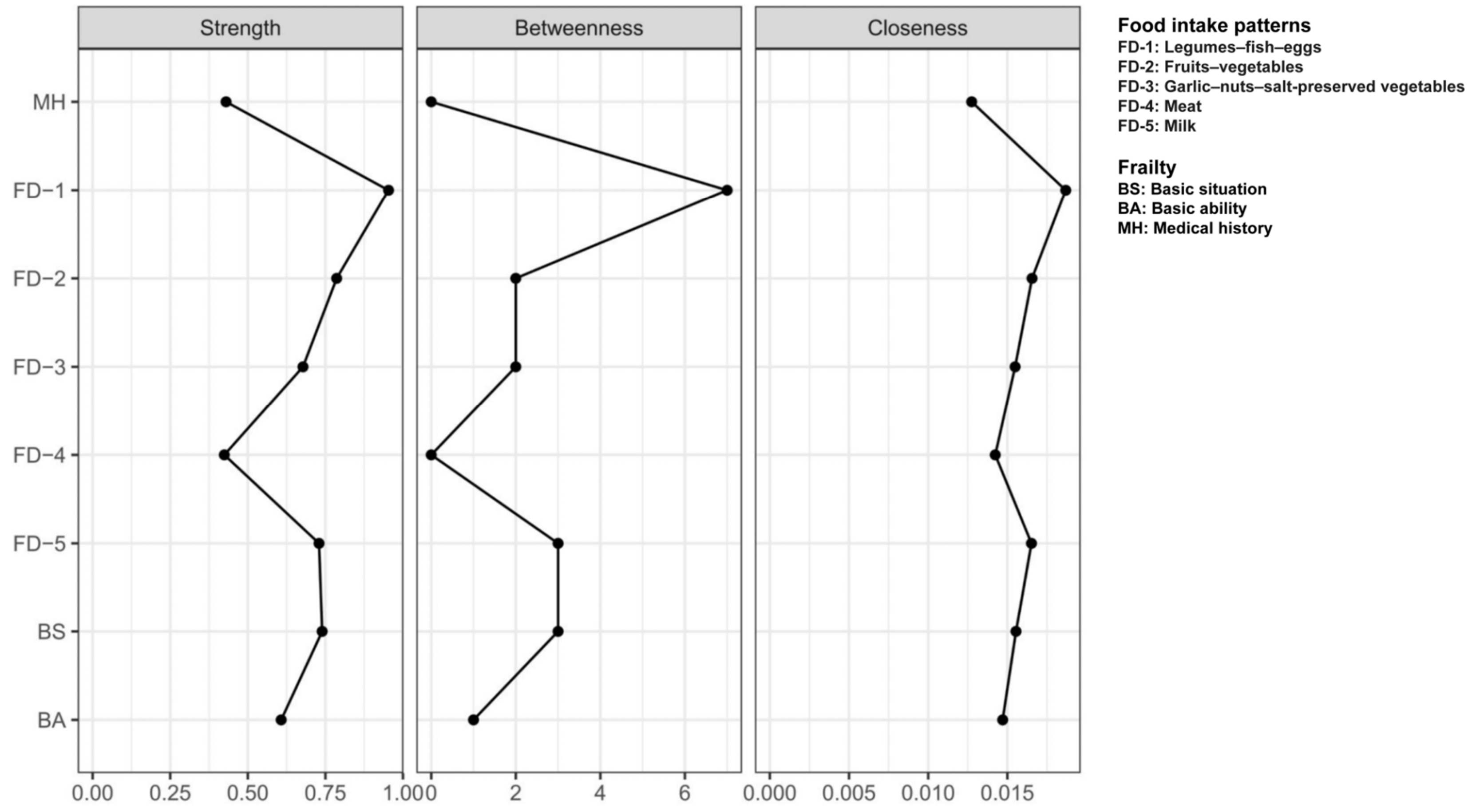

Figure S10. Centrality indicators for the food intake patterns and frailty network among overall older adults (5-factor solution).

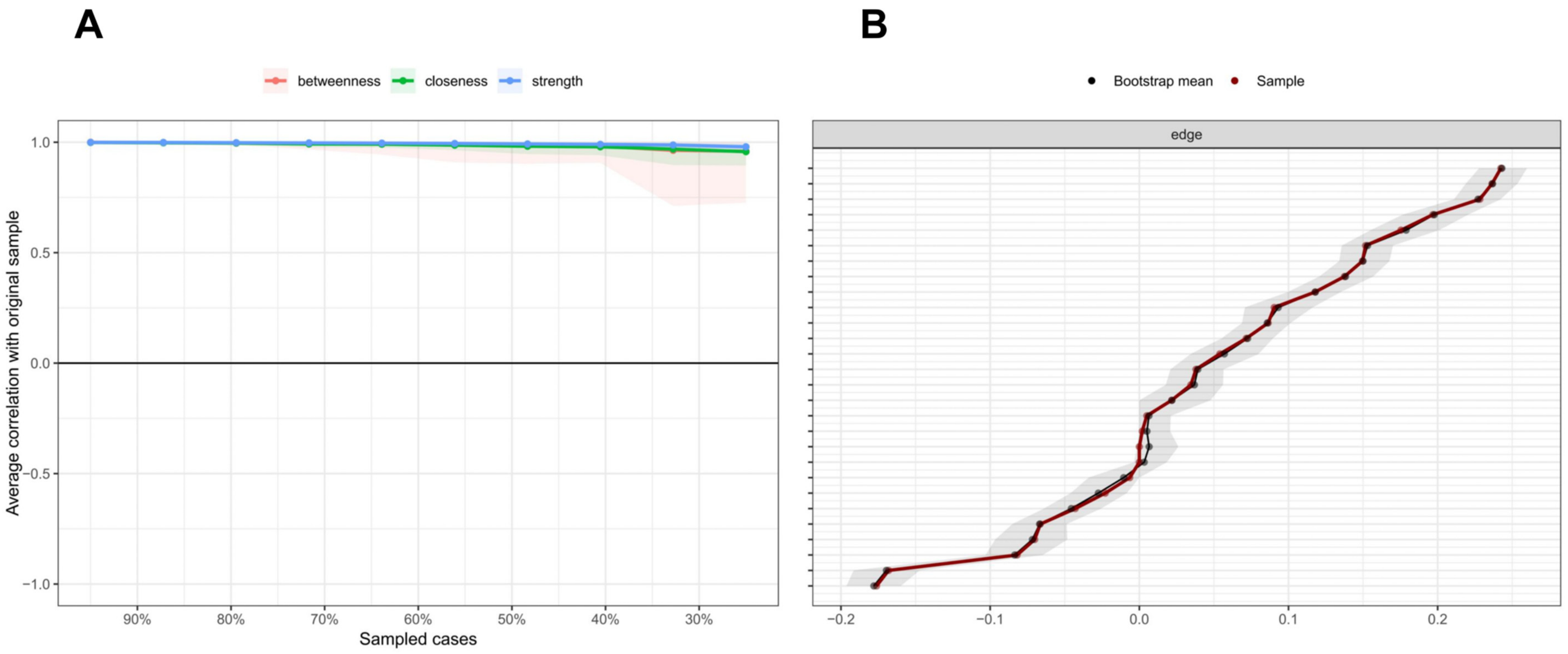

Figure S11. Analysis of network stability and edge-weight accuracy (5-factor solution). A: Centrality stability analysis for the overall older adult population; B: Edge-weight accuracy analysis for the overall older adult population. (In panel A, the curve represents the correlation between centrality measures re-estimated after case exclusion via bootstrap and the original sample centrality measures; in panel B, the gray area indicates the 95% confidence interval estimated by non-parametric bootstrap, the red line represents the original sample edge weight estimates, and black dots represent bootstrap means.) (5-factor solution).
